# MRSA decolonization failure—are biofilms the missing link?

**DOI:** 10.1186/s13756-017-0192-1

**Published:** 2017-03-28

**Authors:** Frank Günther, Brigitte Blessing, Evelina Tacconelli, Nico T. Mutters

**Affiliations:** 1 Department of Infectious Diseases, Heidelberg University Hospital, Im Neuenheimer Feld 324, 69126 Heidelberg, Germany; 2 Division of Infectious Diseases - Department of Internal Medicine I, Tübingen University Hospital, Tübingen, Germany; 3 German Centre for Infection Research (DZIF), Tübingen, Germany

**Keywords:** MRSA, Decolonization, Biofilm, Chlorhexidine, Octenidine, Mupirocin, Polyhexanide, Chloroxylenol, Infection control

## Abstract

**Background:**

Device-associated infections due to biofilm-producing methicillin-resistant *Staphylococcus aureus* (MRSA) have been recently associated with the failure of antibiotic treatment and decolonization measures. The goal of our study was to evaluate the extent to which the formation of biofilms influenced the efficacy of topical decolonization agents or disinfectants such as mupirocin (MUP), octenidine (OCT), chlorhexidine (CHG), polyhexanide (POL), and chloroxylenol (CLO).

**Methods:**

Bacterial killing in biofilms by the disinfectants and MUP was determined as the reduction [%] in metabolic activity determined by a biofilm viability assay that uses kinetic analysis of metabolic activity. The test substances were diluted in water with standardized hardness (WSH) at 25 °C at the standard concentration as well as half the standard concentration to demonstrate the dilution effects in a practical setting. The tested concentrations were: CHG 1%, 2%; OCT 0.1%, 0.05%; PH 0.04%, 0.02%; and CLO 0.12%, 0.24%. A test organism suspension, 1 mL containing ~1 × 10^9^ bacterial cells/mL, and 1 mL of sterile WSH were mixed and incubated for six different exposure times (15 s, 1, 3, 5, 10 and 20 min) after the test substance was added.

Additionally, the bactericidal effects of all substances were tested on planktonic bacteria and measured as the log10 reduction.

**Results:**

The disinfectants OCT and CHG showed good efficacy in inhibiting MRSA in biofilms with reduction rates of 94 ± 1% and 91 ± 1%, respectively. POL, on the other hand, had a maximum efficacy of only 81 ± 7%. Compared to the tested disinfectants, MUP showed a significantly lower efficacy with <20% inhibition (*p* < .05). Bactericidal effects were the greatest for CHG (log10 reduction of 9.0), followed by OCT (7.7), POL (5.1), and CLO (6.8). MUP, however, showed a very low bactericidal effect of only 2.1. Even when the exposure time was increased to 24 h, 2% MUP did not show sufficient bactericidal effect.

**Conclusions:**

Our data provide evidence that OCT and CHG are effective components for disinfection of MRSA-biofilms. On the other hand, exposure to MUP at the standard concentrations in topical preparations did not effectively inhibit MRSA-biofilms and also did not show adequate bactericidal effects. Combining an MUP-based decolonization regimen with a disinfectant such as OCT or CHG could decrease decolonization failure.

## Background

Methicillin-resistant and biofilm-forming *Staphylococcus aureus* (MRSA) isolates have become a common clinical problem [1]. In recent years, MRSA incidences seemed to be decreasing, and the focus of infection control specialists was multidrug-resistant Gram-negative bacteria [2–4]. However, Public Health England recently reported an alarming 26% increase in MRSA bloodstream infections [5]. Although the rise in numbers coincided with the Department of Health’s change in policy on screening for MRSA, from universal to targeted screening, it underlines the fact that MRSA cannot be considered “out of the picture”. The formation of biofilms as a reaction to therapeutic interventions, which can lead to increased antimicrobial resistance and a higher chance of treatment failure, is being increasingly recognized as an infection control problem [6, 7]. Accordingly, treatment and decolonization failure occur more frequently when topical drugs like mupirocin are used against biofilm-forming microorganisms [8–12].

The organization of bacteria into biofilms is the common mode of bacterial survival, since this form increases their ability to withstand antibiotics, disinfectants, and host responses. Biofilm formation is a multifarious, controlled bacterial process that induces many additional functional and phenotypic alterations, including loss of motility, reduced growth rate, increased surface adhesion, as well as an altered susceptibility to the host response [13–17]. An association with biofilm formation has been reported for many hospital-acquired infections, such as urinary tract and catheter-related bloodstream infections as well as infections of implanted medical devices including indwelling catheters, artificial heart valves, orthopedic prostheses, or osteosynthesis materials [7, 18–24]. Colonization with MRSA is associated with a high risk of acquiring an MRSA infection during hospital stays [25, 26]. Decolonization may reduce the risk of MRSA infection in individual carriers and prevent transmission to other patients [25]. However, the most commonly used agent for decolonization, mupirocin, comes with a considerable risk of resistance if widely employed [26]. There have been many other attempts to eradicate carriage, mostly with topical agents, but success rates have not been consistent or applicable to all populations [26], and even mupirocin decolonization success rates can be low [25]. Many international guidelines (i.e., in Germany, Ireland, Netherlands, Slovenia) already state that attempts at decolonization are unlikely to be successful in patients with chronic skin conditions, ulcers, or in-dwelling catheters [27–30]. Some studies, however, showed that decolonization can be effective in patients with lines and catheters [25] and that the inability to decolonize was most closely associated with failure to use a standardized decolonization protocol [31]. However, to our knowledge, no study has been able to identify a consistent subgroup of patients at higher risk for decolonization failure. Perhaps, the focus has been on the wrong variable in the equation, and the reason for decolonization failure is not the patient but the bacterium and its biofilm-forming capacities. Alarmingly, one study on biofilm formation among MRSA nasal carriers showed that all of the isolated MRSA had the ability to form biofilms [7]. The goal of our study, therefore, was to evaluate the extent to which MRSA biofilms are influenced by the use of the topical decolonization antibiotic MUP and the widely used topical disinfectants, i.e., octenidine (OCT), chlorhexidine (CHG), polyhexanide (POL), and chloroxylenol (CLO).

## Methods

### Bacterial isolates

To ensure practical relevance, clinical MRSA isolates, as well as American Type Culture Collection (Manassas, VA, USA; ATCC®) control strains, were tested in this study. Clinical isolates were recovered as follows: screening swab samples were inoculated on Columbia 5% sheep blood agar plate (BD Diagnostics, Sparks, USA) and chromogenic plates for MRSA detection (ChromAgar MRSA II, BD) and incubated under aerobic conditions for 48 h at 36 °C. If growth on chromogenic plates was detected, identification by matrix-assisted laser desorption ionization-time-of-flight mass spectrometry (MALDI-TOF MS) (Bruker Daltonics, Bremen, Germany) was performed [32]. Agglutination with Pastorex® StaphPlus (Alere, Jena, Germany) was performed to confirm *S. aureus* growth. Susceptibility testing was performed by VITEK2 (bioMérieux) and results were interpreted according to EUCAST breakpoints. Biofilm-forming capacities of each isolate were determined by the crystal violet staining technique (data not shown). Six representative isolates with significant biofilm-forming capacity compared to the standard disinfectant efficacy test isolate ATCC® 6538™, according to ATCC® product sheet, were selected and used for further testing.

### Preparation of antimicrobials and neutralizer

For standardization of experimental conditions, in each experiment, water of standardized hardness (WSH) was prepared with a total hardness of 300 ppm (CaCO_3_) with 0.119 g/l magnesium chloride (Carl Roth, Karlsruhe, Germany), 0.277 g/l calcium chloride (Carl Roth), and 0.28 g/l sodium hydrogen carbonate (Carl Roth). WSH at a pH of 7.0 ± 0.2 at 25 °C was used as a diluent. Tryptic soy broth (TSB) containing lecithin, Tween 80, histidine, and sodium thiosulfate neutralizing agent (all from Merck Millipore, Darmstadt, Germany) (LTHTh) was used in both treatment and control groups, immediately following disinfection according to the manufacturer’s instructions. The media and neutralizers used in this study were approved for effective neutralization of the applied disinfectants prior to the experiments (data not shown).

### Biofilm viability assay

The MRSA isolates were cultured on Columbia blood agar plates at 37 °C for 12 h. Bacterial killing in biofilms was determined as reduction [%] in metabolic activity using a kinetic biofilm viability assay as previously described [33]. Briefly, the test substances were diluted in WSH at 25 °C at the standard concentration as well as half the standard concentration to demonstrate the dilution effects in a practical setting. For testing of antimicrobial effects on the bacterial metabolic activity in biofilms, the antimicrobial substances were diluted in WSH at standard working concentrations 0.05% and 0.1% (w/v) for OCT (TCI, Eschborn, Germany), 1% and 2% (w/v) for CHG (Sigma Aldrich, Taufkirchen, Germany), 0.02% and 0.04% (w/v) for POL (Fagron, Barsbuettel, Germany), 0.12% and 0.24% (w/v) for CLO (Sigma Aldrich), and 1% and 2% (w/v) for MUP (Fagron). Each preparation was applied to the prepared biofilms for different exposure times of 15 s, 1, 3, 5, 10 and 20 min at 37°Cfor the OCT, CHG, POL, and CLO solutions. Prolonged exposure times of up to 3.5 h were used for MUP to analyze the different mode of action of this substance. To take into account the different mode of action of MUP compared to disinfectants, extended exposure times of up to 3.5 h were used for this substance adapted to simplified testing protocols for determination of bactericidal activity on *Staphylococcus aureus* isolates as previously described [34]. After exposure, the remaining metabolic activity in the biofilm was measured. Biofilms exposed to WSH alone (without supplements) served as a control for 100% viability or 0% inhibition. The bacterial killing by the disinfectants in the biofilms was determined as the reduction [%] in metabolic activity as compared to the untreated controls.

### Live/dead staining of biofilms

To analyze the killing effects on the bacterial cells in biofilms, biofilms were cultured on glass coverslips (Carl Roth). After incubation, biofilms were washed twice in a 0.9% NaCl solution. Then, 100 μL M63 minimal medium consisting of 0.015 M ammonium hydrogen sulfate (Carl Roth), 0.1 M potassium dihydrogen sulfate (Sigma-Aldrich, Steinheim, Germany), 1.8 μM iron sulfate heptahydrate (Carl Roth), 1 mM magnesium sulfate heptahydrate, 2 ml/L glycerol (VWR Chemicals, Darmstadt, Germany) and 1 g/L casein hydrolysate standard (Carl Roth)containing disinfectants in different concentrations was added, and the biofilms were incubated for up to 2 h at 37 °C. After incubation, biofilms were washed twice in 0.9% NaCl solution and stained using the LIVE/DEAD BacLight Bacterial Viability Kit (Molecular Probes, Leiden, Netherlands) according to the manufacturer’s instructions. Stained biofilms were analyzed after mounting on object slides by using a BZ 8100 fluorescence microscope (Keyence, Neu-Isenburg, Germany) in the green fluorescent band for Syto 9 staining of dead and living bacterial cells, and in red fluorescent band for selective propidium iodide staining of dead cells or cells with disturbed cell integrity; therefore, yellow color in the overlay image is indicative of dead bacteria in the biofilm.

### Determination of bactericidal activity

For determination of bactericidal effects, the antimicrobial substances were diluted in WSH at standard working concentrations 0.1% (w/v) for OCT, 2% for CHG, 0.04% (w/v) for POL, 0.24% (w/v) for CLO, and 2% (w/v) for MUP, and at least 1x10^9^ bacterial cells were added for different exposure times. Each sample was tested for the surviving bacterial count by membrane filtration of the disinfectant solution through a 0.45-μm nitrocellulose membrane (Sartorius Stedim, Göttingen, Germany) followed by rinsing three times using 100 ml of a NaCl-peptone solution (Becton Dickinson, Heidelberg, Germany) for removal of remaining disinfectant. Afterwards, the filters were transferred to Caso Agar containing LTHTh as a neutralizer (Merck Millipore, Darmstadt, Germany). The neutralization and washing steps were validated for effective neutralization of the tested disinfectants prior to this study (data not shown). The media were incubated for 48 h at 37 °C and then checked for microbial growth. Colony forming units (CFUs) were counted and the Log10 reduction factor (LRF_10_) was calculated compared to the untreated controls as a measure of the bactericidal effect.

### Statistics

For descriptive purposes, arithmetic mean value, standard deviation, median, interquartile range, and cumulative frequencies were calculated as appropriate. *P* values of ≤ .05 were considered statistically significant. Statistical analysis was performed using the SPSS ver. 21.0 statistical package (SPSS, Chicago, IL).

## Results

**Fig. 1:**
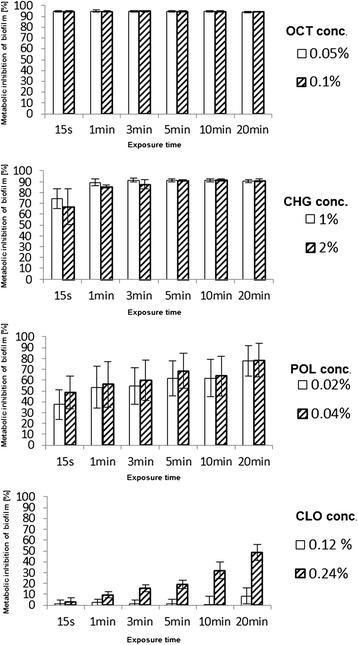
Disinfectant efficacy on MRSA-biofilms Reduction of metabolic activity (%) in bacterial biofilms ± standard deviation (SD) after disinfectant treatment at different concentrations and for varying exposure times, determined by a kinetic metabolic assay

The effects of the different disinfectants on metabolic activity in established MRSA biofilms were tested using a kinetic metabolic assay (Fig. 1) [33]. OCT showed moderate efficacy in inhibiting microbial metabolic activity with 94 ± 1% inhibition after only 15 s of exposure and at a concentration of 0.05%. The overall efficacy of OCT on MRSA biofilm inhibition was not significantly changed due to modifications of the concentration used, ranging from 0.05% to 1%, or due to modifications of the exposure time, ranging from 15 s to 20 min. The CHG solution showed a efficacy of 91 ± 1% in inhibiting microbial metabolic activity after 3 min of exposure to a 1% CHG solution. Furthermore, after 1 min of exposure, there was no significant increase in efficacy of CHG on MRSA biofilms for the tested concentration range. POL, in contrast, yielded a lower overall medium efficacy of inhibition of metabolic activity in biofilms of 65.4 ± 11%, while the efficacy was strongly dependent on the applied exposure time. CLO showed the lowest efficacy of 15.8 ± 27%. Additionally, the efficacy of CLO was strongly dependent on the applied exposure time and concentration.

**Fig. 2:**
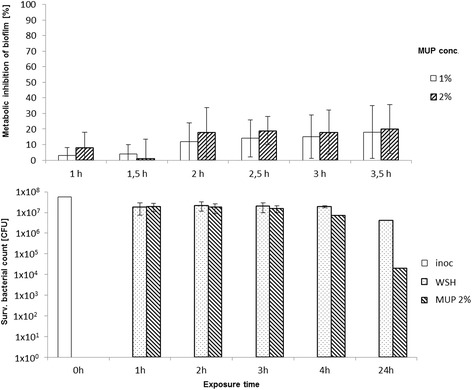
MUP efficacy on MRSA-biofilms and planktonic bacteria Upper panel: Reduction of metabolic activity (%) in bacterial biofilms ± SD after MUP treatment at different concentrations and varying exposure times, determined by a kinetic metabolic assay. Lower panel: Bactericidal activity of a 2% MUP solution on planktonic MRSA, shown as reduction of cell counts [CFU] compared to untreated controls in WSH

On the other hand, the antibiotic MUP showed detectable efficacy in inhibiting metabolic activity in MRSA biofilms after short exposure times (Fig. 2). To rule out that the lack of efficacy is caused by the different mode of action of MUP, namely the inhibition of bacterial protein synthesis, the exposure times for MUP on the MRSA biofilms were extended to up to 3.5 h. Even after 3.5 h of exposure to 2% MUP solution, the level of metabolic inhibition of the bacteria in the MRSA biofilms did not exceed 20%, while significant inhibition (*p* < 0.05) was reached after at least 2 h of. Taken together, 2% MUP showed between 1 and 20% inhibition of the metabolic activity of MRSA biofilms after exposure times of up to 3.5 h (Fig. 2).

**Fig. 3:**
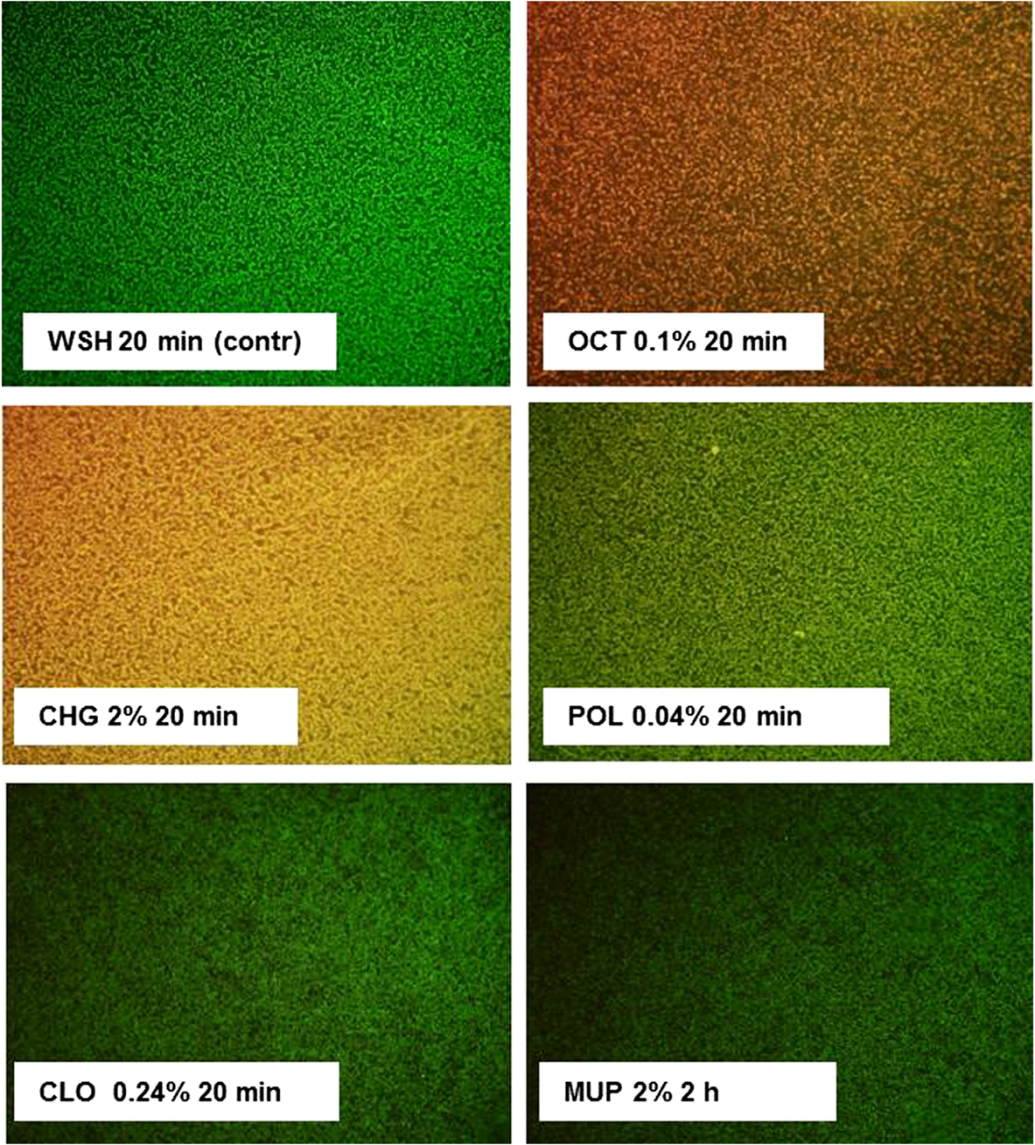
Killing and permeation of MRSA-biofilms by antimicrobials Live/dead-staining using Syto 9 and propidium iodide stains of MRSA biofilms after exposure to WSH, 0.1% OCT, 2% CHG, 0.24% POL, 0.24% CLO, and 2% MUP for varying exposure times. Green: bacteria in biofilm; red: bacterial cell death or defective bacterial cell integrity; yellow: dead bacteria in biofilm

Exposure of biofilms to OCT and CHG solutions led to extensive damage of bacterial cell integrity as indicated by positive propidium iodide staining (Fig. 3), whereas the viability of bacteria in biofilms and the cell integrity of the same strain and maturation state were only weakly affected by POL and CLO and only slightly affected by exposure to 2% MUP for 2 h, similar to that seen in the exposure to WSH only. Additionally, no restrictions of biofilm permeation could be detected for the OCT- and CHG-mediated biofilm killing effects as indicated by the fact that the damage to the biofilm was not restricted to the outside layer.

Regarding the bactericidal effects, each of the four tested disinfectants, OCT, CHG, POL, and CLO, reached a LRF_10_ using planktonic MRSA of at least 4 after 1 h of exposure. In contrast, exposure of MRSA to a 2% MUP solution yielded an LRF_10_ of 0.11 ± 0.2 after 1 h and 2.3 ± 1.8 after 24 h of exposure (Fig. 2).

## Discussion

Our data provide evidence that OCT and CHG are effective components for disinfection of MRSA biofilms in vitro and that exposure to MUP at the standard concentrations in topical preparations up to 2% does not effectively inhibit the metabolic activity of MRSA biofilms, even after the prolonged exposure of 3.5 h. The limited efficacy of MUP against the bacteria in biofilms has previously been described [35]. The biofilm probably provides a physical barrier for MUP so that only insufficient concentrations are reached in the bacteria themselves. Therefore, biofilm formation could influence development of resistance since bacteria in biofilms simply might have more time to adapt to low concentrations of MUP. However, further studies are needed to evaluate actual interactions of MUP with bacterial biofilms.

Nevertheless, our results show that MUP did not have significant bactericidal effects even on planktonic bacteria. In conclusion, MUP has mainly bacteriostatic effects, i.e., inhibition of bacterial growth and reproduction, and only minor effects when bacteria are in biofilms. Biofilms might be the missing link in understanding the rapid development of resistance when MUP-based regimens are used routinely among general inpatient populations.

One limitation of our study is that we cannot estimate the fraction of MRSA strains that are strong biofilm formers. However, this issue should be addressed in future studies. As previously mentioned, one study on 810 nasal carriers of *S. aureus* and MRSA showed that all isolated MRSA strains were biofilm formers; however, only 34.6% were medium to strong biofilm producers [7]. The larger the fraction of strong biofilm formers among MRSA strains, the more impact a change in the decolonization regimen will have.

## Conclusions

The standard MRSA decolonization protocols at referral at our health care facility consist of 5 days of MUP treatment as nasal ointment and additional antiseptic washing of body and daily change of bed linen. Our data suggest that combining an MUP-based decolonization regimen with a disinfectant such as OCT or CHG could increase the efficacy of decolonization in patients colonized with biofilm-forming MRSA. In patients who failed the first decolonization attempt, one possible approach could be to change the 5 days of MUP treatment of the standard decolonization regimen to (1) two days of primary disinfection with either OCT or CHG as a nasal ointment followed by (2) three days of treatment with MUP for suppression of re-colonization. Obviously, this new regimen needs to be evaluated as randomized-controlled trial. Decolonization studies using OCT already show good decolonization rates (67%); however, to our knowledge, no long-lasting effects with regards to re-colonization have yet been reported [36].

## Abbreviations

CFU: Colony forming unit
CHG: Chlorhexidine
CLO: Chloroxylenol
LTHTh: Lecithin, Tween 80, histidine, and sodium thiosulfate neutralizing agent
MRSA: Methicillin-resistant *Staphylococcus aureus*
MUP: Mupirocin
OCT: Octenidine
POL: Polyhexanide
TSB: Tryptic soy broth
WSH: Water with standardized hardness
